# Opinion Formation in the World Trade Network

**DOI:** 10.3390/e26020141

**Published:** 2024-02-05

**Authors:** Célestin Coquidé, José Lages, Dima L. Shepelyansky

**Affiliations:** 1 Équipe de Physique Théorique, Institut UTINAM, Université de Franche-Comté, CNRS, 25000 Besançon, France; 2 Laboratoire de Physique Théorique, Université de Toulouse, CNRS, UPS, 31062 Toulouse, France; dima@irsamc.ups-tlse.fr

**Keywords:** international trade, complex networks, opinion formation model

## Abstract

We extend the opinion formation approach to probe the world influence of economical organizations. Our opinion formation model mimics a battle between currencies within the international trade network. Based on the United Nations Comtrade database, we construct the world trade network for the years of the last decade from 2010 to 2020. We consider different core groups constituted by countries preferring to trade in a specific currency. We will consider principally two core groups, namely, five Anglo-Saxon countries that prefer to trade in US dollar and the 11 BRICS+ that prefer to trade in a hypothetical currency, hereafter called BRI, pegged to their economies. We determine the trade currency preference of the other countries via a Monte Carlo process depending on the direct transactions between the countries. The results obtained in the frame of this mathematical model show that starting from the year 2014, the majority of the world countries would have preferred to trade in BRI than USD. The Monte Carlo process reaches a steady state with three distinct groups: two groups of countries preferring to trade in whatever is the initial distribution of the trade currency preferences, one in BRI and the other in USD, and a third group of countries swinging as a whole between USD and BRI depending on the initial distribution of the trade currency preferences. We also analyze the battle between three currencies: on one hand, we consider USD, BRI and EUR, the latter currency being pegged by the core group of nine EU countries. We show that the countries preferring EUR are mainly the swing countries obtained in the frame of the two currencies model. On the other hand, we consider USD, CNY (Chinese yuan), OPE, the latter currency being pegged to the major OPEC+ economies for which we try to probe the effective economical influence within international trade. Finally, we present the reduced Google matrix description of the trade relations between the Anglo-Saxon countries and the BRICS+.

## 1. Introduction

The process of opinion formation is at the foundation of functioning of democratic societies [1]. The development of social networks, characterized by scale-free properties (see, e.g., [2,3]), makes the investigation and analysis of such a process even more important. Various voter models have been developed for the analysis of opinion formation, as described in [2,4,5,6,7,8,9,10,11,12,13,14]. It is natural to assume that a given voter opinion is influenced or even determined by the opinions of directly linked neighbors, similar to the spin magnetization in the Ising model: if the spins neighboring a spin, namely, a voter, are mainly up-oriented, this spin also turns up, or if the neighboring spins are down-oriented, then the spin turns down. Such an approach to opinion formation on regular lattices and complex networks have been applied in many cases and analyzed in the above cited publications. At the same time, in [1], it was argued that a social elite has significant influence on opinion formation. This feature was taken into account in [15,16] by attributing to each spin a weight proportional to the PageRank probability of each node (or voter, or spin). Such a PageRank probability is determined from the PageRank algorithm applied to the Google matrix of a directed social network or other complex network.

The PageRank algorithm, invented in [17], allows for efficiently computing the eigenvector of the Google matrix of a directed network corresponding to the leading eigenvalue. According to the Perron–Frobenius theorem, the components of this vector are positive and give the stationary probabilities to find a random surfer on a given node after a long series of jumps from node to node on the network. The Google matrix construction is based on a Markov chain over the network with transition probabilities determined by the number of links from a node to the other nodes (see details in [17,18]). The nodes with the highest PageRank probabilities correspond to the most influential nodes in the network. Such nodes were associated with social elite, and it was shown, in agreement with the analysis presented in [1], that they significantly influence the opinion formation on social and other directed networks [15,16,19].

Various properties of real directed networks, their Google matrices with their spectra and eigenstates are described in [20]. Among such complex networks, an interesting example is the World Trade Network (WTN) constructed from the United Nations (UN) Comtrade database [21], which gathers the annual commercial transactions between world countries. The properties of the WTN Google matrix were studied, with details in [22,23,24,25]. An interesting new element of the WTN is that not only does the PageRank vector play an important role, but so does the additional CheiRank vector [26,27], which represents the PageRank vector of the WTN for which the direction of the transactions are inverted. Indeed, the PageRank and CheiRank probability of a node are approximately proportional to the number of ingoing and outgoing links of this node, respectively [18,20]. Usually, e.g., for the World Wide Web (WWW) [17,18], the outgoing links are not very important since they can be easily modified at the node level. However, in the WTN, ingoing and outgoing trade transactions correspond to import or export of a given country and definitely both play very important roles. Thus, both the PageRank and the CheiRank vectors are essential for the analysis of WTN flows [23,24,25].

It is rather natural to apply the approach of opinion formation to the WTN. For the WTN, we consider that a country can have its own opinion to perform a trade with a certain group of countries with one preferred currency (e.g., US dollar, USD) and with another group of countries with another currency (e.g., Chinese yuan, CNY). It is possible to study the opinion evolution of the countries, assuming that initially, a given fraction $f_{i}^{\mathrm{USD}}$ of all the countries have a trade currency preference (TCP) for USD, while the remaining fraction $f_{i}^{\mathrm{CNY}}=1-f_{i}^{\mathrm{USD}}$ initially prefers to trade in CNY. Taking these initial distributions as random, a Monte Carlo iteration step is performed *asynchronously*: for a given country, a new TCP is determined according to a certain linear combination of opinions of its neighbors, and this country possibly adopts the opinion of the majority of its neighbors. This procedure is performed sequentially for all countries picked at random, and at the end of such asynchronous Monte Carlo iterations, we obtain the final TCP distributions and, consequently, the final fractions $f_{f}^{\mathrm{USD}}=1-f_{f}^{\mathrm{CNY}}$ of countries preferring to trade in USD or CNY. Such an approach was applied for the WTN for the years 2010–2020, showing that around 2014, the major fraction of countries would prefer to trade in CNY [28]. It is interesting to note that similar Monte Carlo iterations are used in the models of associative memory, even if the linear condition for spin orientation imposed by neighbors is somewhat different there (see, e.g., [29,30]).

For the WTN, it is also possible to consider the case of three currencies (three possible TCPs), e.g., USD, BRI and EUR, where BRI is a hypothetical currency pegged to the BRICS economies [31]. Here, the TCP of a country is modeled as a spin, which takes its orientation accordingly to the distribution of the three possible TCPs among its neighbors. It was also assumed [31] that for each possible currency, there are core groups of countries that always have a fixed TCP: USD for 5 Anglo-Saxon countries, the *hypothétique* BRI currency for the 5 BRICS and EUR for the kernel of 9 EU countries. The kernel of 9 EU countries was chosen following the economic studies reported in [32,33]. We note that the presence of the core groups with fixed opinion is reminiscent somehow of the case of the Sznajd opinion formation model [6], which captures the known feature of the trade unions’ slogan *united we stand, divided we fall*.

In this paper, we review the opinion formation approach applied to the WTN with two or three currencies. We apply this approach mainly to the case of the currency battle between USD and BRI through the core groups of 5 Anglo-Saxon countries and of the 11 countries that are expected to form the new BRICS+ group from 2024 [34,35]. We also consider other cases with three currencies and with other core groups for the years 2010–2020. We also analyze the effective transactions between the countries belonging to the core groups using the reduced Google matrix (REGOMAX) approach applied to the WTN [25,36].

It should be noted that from the Bretton Woods agreement in 1944 until last year, USD kept the dominant position in international trade [37]. However, recently, there is a clear tendency for certain countries to perform trade in other currencies. For example, Saudi Arabia considers using Chinese yuan (CNY) instead of USD for oil sales to China [38]. Also, CNY has become the most traded foreign currency at the Moscow Exchange [38]. The Brazil–China authorities summit in April 2023 pushed forward the possibility of launching a new currency, BRI, pegged to the five BRICS economies to end the trade dominance of USD [39]. From January 2024, BRICS is expected to expand to six new countries, leading to the appearance of the new BRICS+ group [34,35], whose creation can significantly affect international trade, notably with the hypothetical appearance of its own BRI currency. Thus, the analysis of the WTN with opinion formation approach in the frame of this de-dollarization attempt era is rather interesting and timely.

The article is composed as follows: Section 2 describes the data sets, the opinion formation model on the WTN and the Google matrix construction; Section 3 presents the results of the battle between two currencies, USD and BRI, which are supported by Anglo-Saxon countries and the BRICS+, respectively; Section 4 depicts the trade interactions between these countries obtained in the frame of the REGOMAX algorithm; Section 5 reports the results of the three currencies battle between USD, BRI and EUR and then USD, CNY and OPE (a hypothetical petrocurrency pegged to the major OPEC+ economies); the discussion and conclusion are given in Section 6. Appendix A supports the main results presented in the article.

## 2. Model Description and Data Sets

We use the UN Comtrade database [21], which gathers the yearly volumes of bilateral commercial transactions of about tens of thousands of products. We consider international trade between N=194 countries over the decade 2010–2020. We use the aggregated money matrix element $M_{cc^{\prime }}$ which gives the volume of goods, expressed in USD, exported from the country $c^{\prime }$ to the country *c* during a given year. The total volume of commodities imported by and exported from the country *c* is then $M_{c}=\sum_{c^{\prime }}M_{cc^{\prime }}$ and $M_{c}^{*}=\sum_{c^{\prime }}M_{c^{\prime }c}$, respectively. The total volume of goods exchanged in a given year is $M=\sum_{c}M_{c}=\sum_{c}M_{c}^{*}$. The quantities

(1) $$S_{cc^{\prime }}=\frac{M_{cc^{\prime }}}{M_{c^{\prime }}^{*}}\;\mathrm{and}\;S_{cc^{\prime }}^{*}=\frac{M_{c^{\prime }c}}{M_{c^{\prime }}}$$

give, respectively, the fraction of the total volume exported from the country $c^{\prime }$, which is effectively imported by the country *c*, and the fraction of the total volume imported by the country $c^{\prime }$, which is effectively exported from the country *c*. These quantities measure the relative importance of the country *c* in the exports and the imports of the country $c^{\prime }$. The ImportRank and the ExportRank, i.e.,

(2) $$P_{c}=\frac{M_{c}}{M}\;\mathrm{and}\;P_{c}^{*}=\frac{M_{c}^{*}}{M}$$

quantify, respectively, the relative importance of the imports by and the exports from country *c* with respect to the total volume of goods exchanged in the WTN in a given year.

### 2.1. Asynchronous Monte Carlo Determination of Trade Currency Preferences

Let us consider asynchronous Monte Carlo steps:

*Step 0*—A TCP is randomly assigned to each country. Hence, a given country *c* initially prefers to trade either with the currency ${¢}_{-}$ or with the other ${¢}_{+}$. An initial world distribution of the TCPs is then obtained.

*Step 1*—A country *c* with an initial TCP ${¢}_{0}$, i.e., either ${¢}_{-}$ or ${¢}_{+}$, is picked at random. For this country, we compute the following TCP score

(3) $$Z_{c}=\sum_{c^{\prime }\neq c}\sigma_{c^{\prime }}\left(S_{c^{\prime }c}+S_{c^{\prime }c}^{*}\right)\frac{\left(P_{c^{\prime }}+P_{c^{\prime }}^{*}\right)}{2},$$

where the sum is performed over all the countries $c^{\prime }$, which are economical partners of the country *c*. The $\sigma_{c^{\prime }}$ parameter is equal to −1 if the country $c^{\prime }$ TCP is ${¢}_{-}$ and +1 if the country $c^{\prime }$ TCP is ${¢}_{+}$. The country *c* adopts, then, either the TCP ${¢}_{-}$ if $Z_{c}<0$ or the TCP ${¢}_{+}$ if $Z_{c}\geq 0$. Consequently, the TCP of country *c* possibly changes from the previous step currency ${¢}_{0}$ to the new currency ${¢}_{1}$. A second country is picked at random for which a new TCP-score (3) is computed. Following the same rule as the one used for the first country, the second country TCP possibly changes and so on, until a new TCP-score (3) is computed for the last-picked country, and possibly the TCP of this country changes. At the end of *step 1*, if the world distribution of the TCPs is the same as the *step 0* one, then the Monte Carlo iteration is stopped, otherwise *step 2*, similar to *step 1*, is initiated based on the new TCP world distribution obtained at the end of *step 1*. The asynchronous Monte Carlo iterations stop at *step n*, such that the TCP world distribution obtained at the end of *step n* is the same as the one obtained at the end of the *step n-1*. The system then reaches an equilibrium characterized by a specific world distribution of TCPs.

### 2.2. Google Matrix and Reduced Google Matrix Construction

We follow the procedure detailed in [25] to construct the Google matrix *G* associated with the WTN. This Google matrix [18] encodes the stochastic process describing a random walk on the WTN. The Google matrix element $G_{cc^{\prime }}=\alpha S_{cc^{\prime }}+\left(1-\alpha \right)/N$ encodes the trade interaction between country $c^{\prime }$ and *c*. The probability rate to jump from country $c^{\prime }$ to the node *c* is given by the stochastic matrix elements $S_{cc^{\prime }}$ (1). Hence, the random walker follows the WTN structure with the probability α and can be teleported to any country with a probability of 1−α. Here, the damping factor α=0.85 ensures a unique steady state of the random walk independent of the initial conditions. The steady state is characterized by a Perron vector [18], Ψ, called the PageRank vector [17], defined as GΨ=Ψ. The component $\psi_{c}$ of the PageRank vector Ψ is proportional to the number of times the random walker hits the country *c* during its journey in the WTN.

The component $\psi_{c}$ of the PageRank vector Ψ is proportional to the number of times the random walker forever wandering in the WTN hits the country *c* during its journey. Similarly to the *G* Google matrix, it is useful to consider the $G^{*}$ Google matrix associated with the WTN, of which all the money flows have been inverted [23,24,25]. The Perron vector $\Psi^{*}$, such as $G^{*}\Psi^{*}=\Psi^{*}$, is known as the CheiRank vector of the WTN [26,27]. While the PageRank vector Ψ characterizes ingoing flows (imports), the CheiRank vector characterizes outgoing flows (exports). Let us note that these matrix elements of the *G* and $G^{*}$ matrices characterize the trade flows between the countries also related to probability and entropy flows.

Here, we use the reduced Google matrix (REGOMAX) analysis, borrowed from the quantum scattering theory, already used in [25] and described with details in [36]. Let us consider a set of $N_{r}$ selected countries. Just as the global Google matrix *G* expresses the transactions carried out through the WTN by the *N* world countries (with GΨ=Ψ), the goal of the REGOMAX analysis is to construct a reduced Google matrix $G_{R}$ associated with the $N_{r}$ selected countries (with $G_{R}\Psi_{r}=\Psi_{r}$, where the $\Psi_{r}$ vector gathers the components of the PageRank vector Ψ corresponding to the selected countries). This $G_{R}$ matrix then expresses the effective direct and indirect transactions between these countries. One of the main interests of the REGOMAX analysis is that it effectively takes into account all the information initially contained in the WTN. According to [36], the reduced Google matrix can be split into three components, i.e., $G_{R}=G_{\mathrm{rr}}+G_{\mathrm{pr}}+G_{\mathrm{qr}}$. The $G_{\mathrm{rr}}$ component is the $N_{r}\times N_{r}$ sub-matrix of the matrix *G* corresponding to the $N_{r}$ countries. Hence, the $G_{\mathrm{rr}}$ matrix is filled with the $G_{cc^{\prime }}$ components of the WTN Google matrix *G*, with countries *c* and $c^{\prime }$ belonging to the set of selected countries. While the $G_{\mathrm{rr}}$ matrix describes the direct trade links between the selected countries, the $G_{\mathrm{pr}}+G_{\mathrm{qr}}$ matrix describes the indirect trade links between the selected countries but passing through the rest of the global network made up of the other countries. The $G_{\mathrm{pr}}∼\Psi_{r}e^{T}$ matrix, where $e^{T}=\left(1,1,\ldots ,1\right)$, is uninteresting, as it roughly contains only information about the reduced PageRank vector $\Psi_{r}$. The remaining component $G_{\mathrm{qr}}$ encodes the possible hidden commercial links between the $N_{r}$ selected countries.

A qualitative description of the various features of the REGOMAX algorithm and its applications to the MetaCore network of protein–protein interactions is given in [40].

## 3. Battle of Two Currencies: USD versus BRI

In this Section, we discuss the results obtained for the currency battle between a set of 5 countries of the Anglo-Saxon world (named hereafter ANGL) and the set of the 11 BRICS+ countries. The list of these countries is given in Table 1. We use the WTN built from the UN Comtrade database [21] for the years 2010–2020. Of course, the BRICS+ was not yet formed at that epoch, but we model it as a *hypothétique* group to characterize its potential influence nowadays and in the future. We assume that the countries of each of these two core groups, ANGL and BRICS+, always perform trade in USD and BRI, respectively. Thus, their corresponding Ising spins $\sigma_{c}$ are always oriented down, i.e., $\sigma_{c}=-1$, for USD and up, i.e., $\sigma_{c}=+1$, for BRI. Then, we consider an initial configuration of random up and down spin orientations for the other 178 countries (194 countries minus the 16 countries listed in Table 1), mimicking the world distribution of the initial TCPs (*step 0* of the asynchronous Monte Carlo procedure described in Section 2.1). The initial fraction of spins oriented down is $f_{i}^{\mathrm{USD}}$, and the initial fraction of spins oriented up is $f_{i}^{\mathrm{BRI}}=1-f_{i}^{\mathrm{USD}}$. As the TCPs of the 16 countries belonging to the core groups (ANGL and BRICS+) are fixed, we perform asynchronous Monte Carlo iterations on the remaining 177 countries (see Section 2.1). After each step, the TCP-score world distribution (3) is computed until the convergence to a steady-state configuration of the TCPs over the world countries is achieved. For a given initial fraction $f_{i}^{\mathrm{USD}}$ of countries with a TCP for USD, we performed the asynchronous Monte Carlo iterations from $N_{\mathrm{conf}}=10^{4}$ different initial random spin configurations. The final fraction of spins oriented down, i.e., the final fraction of countries preferring USD, is $f_{f}^{\mathrm{USD}}$, and the final fraction of spins oriented up, i.e., the final fraction of countries preferring BRI, is $f_{f}^{\mathrm{BRI}}=1-f_{f}^{\mathrm{USD}}$. The convergence to a steady-state orientation of all the spins takes place after τ steps (one step corresponds to the 177 asynchronous iterations and computations of (3) for the 177 spins).

**Figure 1:**
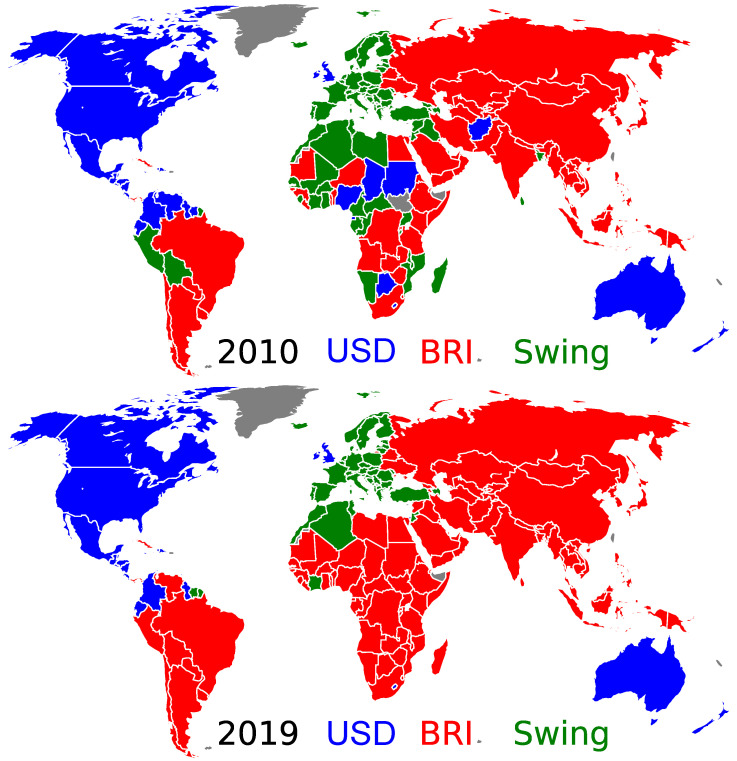
World distribution of the trade currency preferences for the years 2010 (**top**) and 2019 (**bottom**). The countries belonging to the USD group and the BRI group are colored in blue and red, respectively. Those belonging to the swing group are colored in green. Countries colored in gray have no trade data reported in the UN Comtrade database for the considered year [21]. The world distribution of trade currency preferences for 2012, 2014, 2016, 2018 and 2020 are presented in Figure A1.

**Figure 2:**
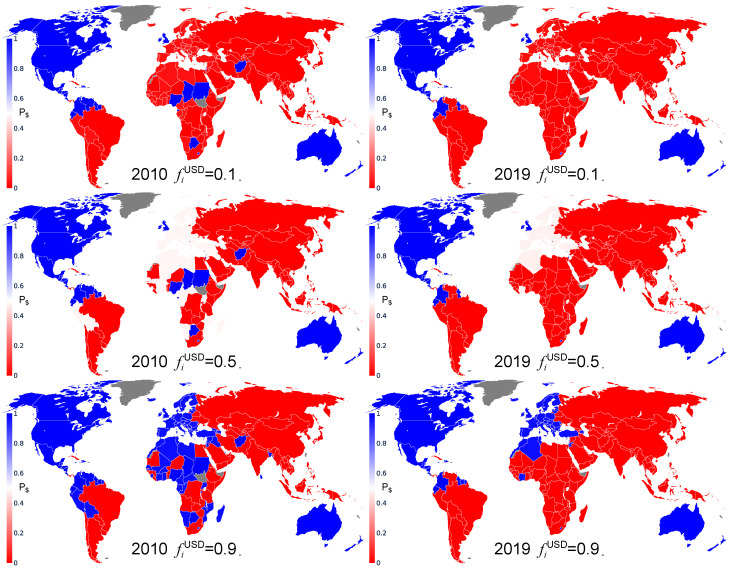
World distribution of the probability $P_{\$}$ that a country chooses USD as its trade currency for 2010 (**left**) and 2019 (**right**), and for $f_{i}^{\mathrm{USD}}=0.1$ (**top**), 0.5 (**center**) and 0.9 (**bottom**). The colors range from red for countries which always have a TCP for BRI ($P_{\$}=0$) to blue for countries that always have a TCP for USD ($P_{\$}=1$). Countries colored in gray have no trade data reported in the UN Comtrade database for the considered year [21].

**Figure 3:**
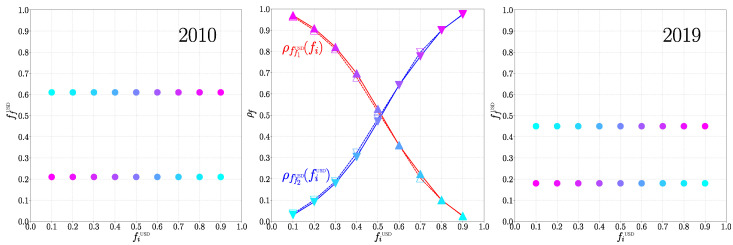
Final fraction $f_{f}^{\mathrm{USD}}$ of countries with a trade currency preference for USD versus the initial fraction $f_{i}^{\mathrm{USD}}$ of these countries for years 2010 (left panel) and 2019 (right panel). There are two possible final fractions for each considered year: $f_{f_{1}}^{\mathrm{USD}}=0.21$ and $f_{f_{2}}^{\mathrm{USD}}=0.61$ in 2010, and $f_{f_{1}}^{\mathrm{USD}}=0.18$ and $f_{f_{2}}^{\mathrm{USD}}=0.45$ in 2019. The color of the points represents the ratio of the Monte Carlo process with the corresponding final state $\rho_{f_{f}}\left(f_{i}\right)$, low ratio in cold blue and high ration in violet. The central panel shows the evolution of $\rho_{f_{f}}\left(f_{i}\right)$ with $f_{i}$. The red (blue) curve and the up (down) triangles denote the minimal (maximal) final state. The full (empty) symbols correspond to the year 2019 (2010).

**Figure A1:**
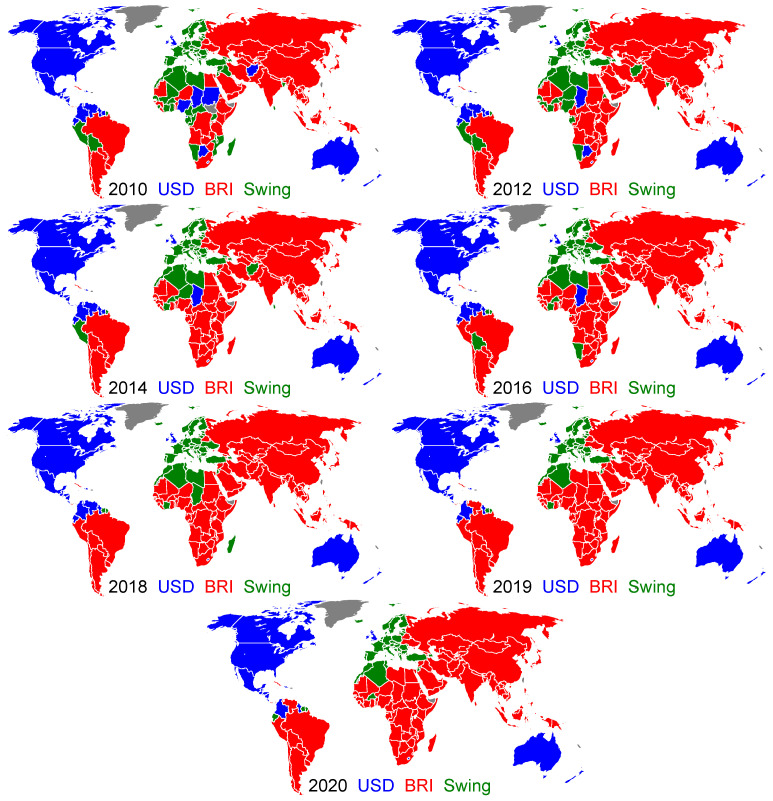
World distribution of the trade currency preferences for the years 2010, 2012, 2014, 2016, 2018, 2019 and 2020. The countries belonging to the USD group and the BRI group are colored in blue and red, respectively. Those belonging to the swing group are colored in green. Countries colored in gray have no trade data reported in the UN Comtrade database for the considered year [21].

**Figure A2:**
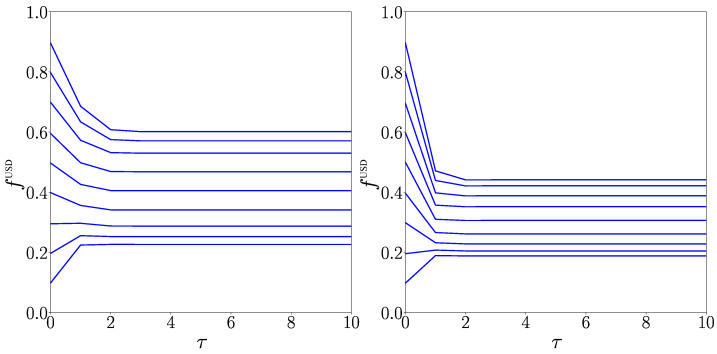
Evolution of the fraction of countries preferring USD, $f^{\mathrm{USD}}$, with the number of step τ of the asynchronous Monte Carlo procedure (Section 2.1). The fraction $f^{\mathrm{USD}}$ is averaged over $10^{4}$ random initial configurations. The left (right) panel concerns the 2010 WTN (2019 WTN).

The main obtained results are presented in Figure 1, Figure 2 and Figure 3, with related Figure A1 and Figure A2 in Appendix A. A rapid convergence of the asynchronous Monte Carlo iterations (see Section 2.1) is shown in Figure A2. Indeed, the convergence is reached after approximately τ=4 steps (Figure A2). In fact, only two stable values of the final fraction $f_{f}^{\mathrm{USD}}$ exist: $f_{f}^{\mathrm{USD}}=0.21$ and 0.61 in 2010; and $f_{f}^{\mathrm{USD}}=0.18$ and 0.45 in 2019 (see Figure 3). Although these two stable values are independent of the initial fraction $f_{i}^{\mathrm{USD}}$, the probability $\rho_{f_{f}^{\mathrm{USD}}}\left(f_{i}\right)$ to reach a given stable fraction value $f_{f}^{\mathrm{USD}}$ changes with the initial fraction $f_{i}^{\mathrm{USD}}$, as shown in Figure 3. The two final possible outcomes for $f_{f}^{\mathrm{USD}}$ evolve from year to year. The existence of two final attractors $f_{f}^{\mathrm{USD}}$ for the asynchronous Monte Carlo iterations implies that certain countries, outside of the ANGL and BRICS+ core groups, always keep their TCP independent of the initial fraction $f_{i}^{\mathrm{USD}}$. At the same time, there are countries, hereafter named swing countries, that change their TCP depending on the $f_{i}^{\mathrm{USD}}$ value. Thus, we have three groups that are the USD group gathering the countries of the ANGL group and the countries with USD as final TCP, the BRI group gathering the BRICS+ and the countries with BRI as final TCP, and the swing group. The world distribution of these groups is shown in Figure 1 for the years 2010 and 2019 (the world maps for other years in the range 2010–2020 are shown in Figure A1). There is a striking evolution from 2010 to 2019: the number of swing countries is significantly reduced, and almost all Latin American, African and Asian countries have a TCP for BRI in 2019. In 2019, the swing countries only comprise the EU countries, Norway, Former Yugoslavia countries, Albania, Moldavia, Azerbaijan, Turkey, Liban, Tunisia, Algeria, Morocco, Ivory Coast and Suriname. According to the Figure A1, such a transition takes place around the year 2016.

For the swing countries, the probability to have one or the other TCP depends on the initial fraction $f_{i}^{\mathrm{USD}}$, as shown in Figure 2. For $f_{i}^{\mathrm{USD}}=0.9$, all the swing countries in 2010 and 2019 prefer to trade with USD with the probability close to unity (but the number of countries with a TCP for USD is reduced from 2010 to 2019). However, for $f_{i}^{\mathrm{USD}}=0.5$, in 2010 and 2019, all the European countries have a probability of about $P_{\$}≃0.5$ to choose USD as trade currency. For $f_{i}^{\mathrm{USD}}=0.1$, practically all the countries would prefer to trade with BRI, with a probability close to unity ($P_{\$}≃0$) on 2019 (besides countries belonging to the ANGL group, only the Central American countries, Colombia and Ecuador would keep a TCP for USD).

**Figure 4:**
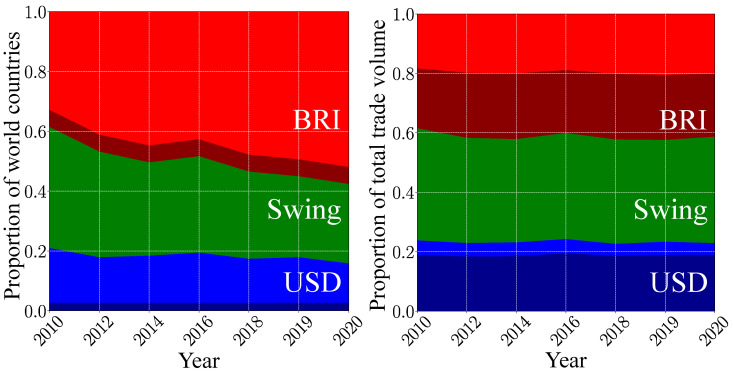
Time evolution of the size of the trade currency preference groups. The width of a given band corresponds to the corresponding fraction of world countries in a TCP group (**left panel**) and to the corresponding fraction of the total trade volume generated by this group (**right panel**). The USD group is colored in blue, the BRI group in red, and the swing group in green. Within the BRI (USD) group, the proportion corresponding to the BRICS+ (the ANGL countries) is shown in dark red (dark blue).

These results, obtained in the frame of our mathematical model, show that the hypothetical BRI currency will become dominant in present-day world trade. This is corroborated by the proportion of countries belonging, respectively, to the USD, the BRI and the swing groups (see Figure 4). The BRI group grows from about 38% of the world countries in 2010 to 58% in 2020. As the USD group only slightly decreases during this decade, the BRI group has mainly captured countries from the swing group. However, these captured countries have a somewhat small economic weight, as the relative proportions of the total trade volume corresponding to these three groups stay stable during the last decade. Hence, in 2020, the BRI group represents 41% of the total trade volume, the swing group 36% and the USD group the remaining 23%. For the USD group, the major trade volume is associated with the ANGL countries. By contrast, the BRICS+ only represent about a half of the total trade volume proportion of the BRI group. Contrarily to the USD group, the BRI currency is able to influence countries with a non-negligible economic weight way beyond the perimeter of the BRICS+.

**Figure A3:**
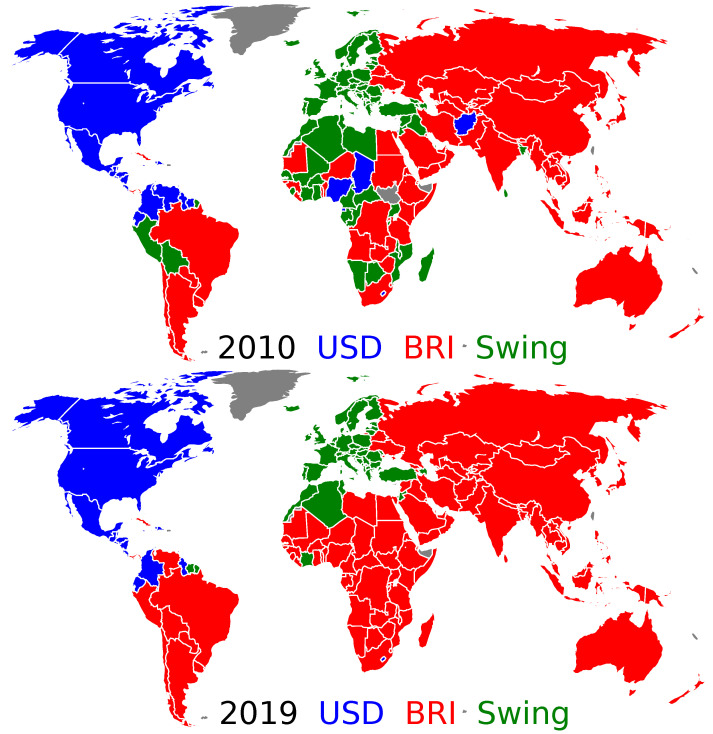
Same as Figure 1 but with the ANGL group only containing the USA.

**Figure A4:**
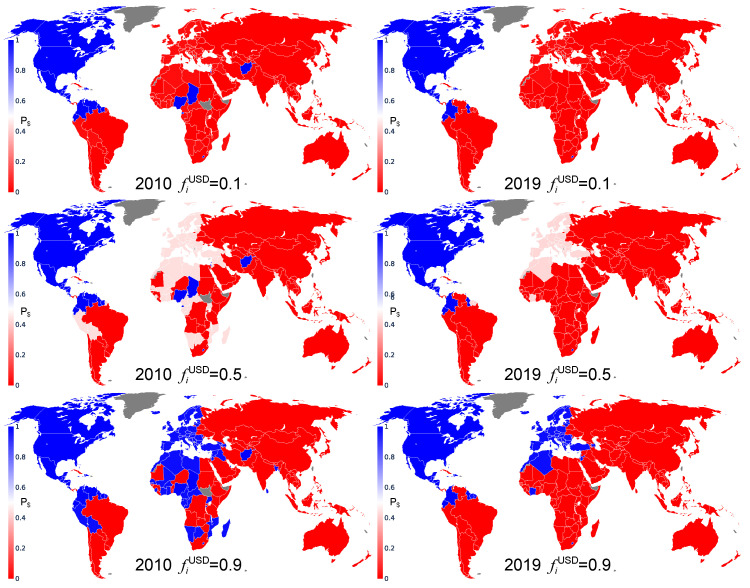
Same as Figure 2 but with the ANGL group only containing the USA.

We have previously considered the ANGL group formed by five countries of the Anglo-Saxon world. In order to analyze how the USA is influential among the other Anglo-Saxon countries, we consider the case where the ANGL group only contains the USA, the remaining Anglo-Saxon countries now being free to choose their TCP. We keep, nonetheless, the BRICS+ group unchanged. The results corresponding to this change are shown in Figure A3 and Figure A4. From Figure A3, we see, both in 2010 and 2019, that among Anglo-Saxon world countries, Australia and New Zealand firmly adopt the BRI trade preference, UK enters the swing group and only Canada keeps USD as its TCP. The evaluation of the final TCP probability for different initial $f_{i}^{\mathrm{USD}}$ values is shown in Figure A4. The world maps are similar to those of Figure 2, with the main difference that Australia and New Zealand always have a TCP for BRI and that UK prefers USD for $f_{i}^{\mathrm{USD}}=0.9$, and prefers as the other European countries BRI for $f_{i}^{\mathrm{USD}}=0.5$ and 0.1 (resulting in $f_{f}^{\mathrm{USD}}≃0.58$ and 0.16, respectively). These results show that the choice of the UK’s TCP is similar to those of the EU countries, as one could expect for the considered pre-Brexit period, and that Australia and New Zealand have very strong economic ties with the BRICS+ countries.

Also, the comparison of Figure 1 with Figure 4 in [28] (an article devoted to the battle of USD vs. CNY in international trade) shows that in the BRICS+, the dominant economic role is played by China (see the similarity of the distributions of the swing countries in the two figures).

**Figure A5:**
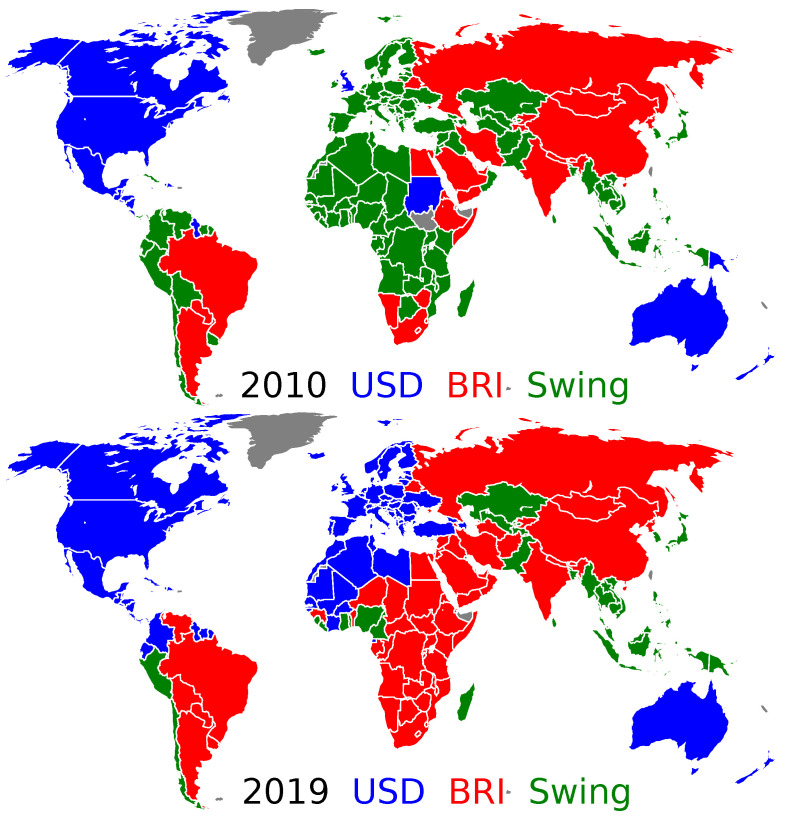
Same as Figure 1 but for $\left(P_{c^{\prime }}+P_{c^{\prime }}^{*}\right)=1$ in the TCP-score (3). World distribution of the trade currency preferences for the years 2010 (top) and 2019 (bottom), taking $f_{i}^{\mathrm{USD}}=0.9$ as the initial fraction of countries preferring USD. The countries belonging to the USD group and the BRI group are colored in blue and red, respectively. Those belonging to the swing group are colored in green. Countries colored in gray have no trade data reported in the UN Comtrade database for the considered year [21].

**Figure A6:**
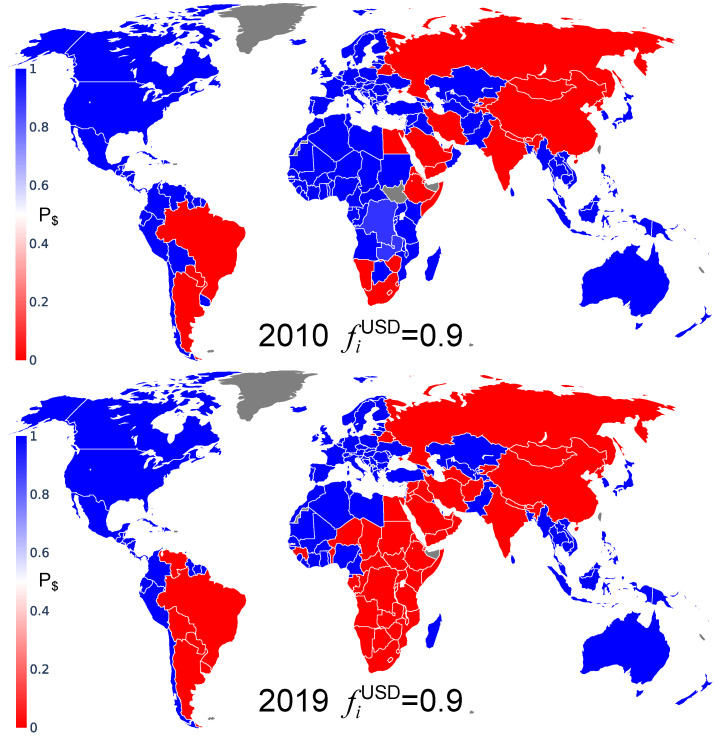
Same as Figure 2 but for $\left(P_{c^{\prime }}+P_{c^{\prime }}^{*}\right)=1$ in the TCP-score (3). World distribution of the probability $P_{\$}$ that a country chooses USD as its trade currency for 2010 (**top**) and 2019 (**bottom**), and for $f_{i}^{\mathrm{USD}}=0.9$. The colors range from red for countries that always have a TCP for BRI ($P_{\$}=0$), to blue for countries that always have a TCP for USD ($P_{\$}=1$). Countries colored in gray have no trade data reported in the UN Comtrade database for the considered year [21].

**Figure A7:**
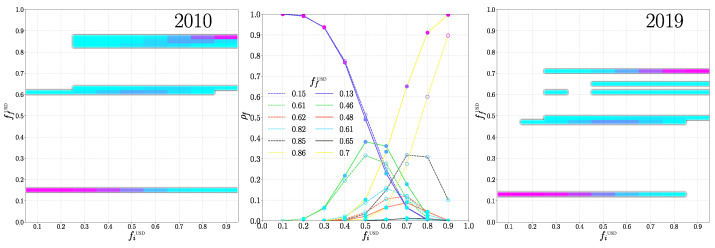
Same as Figure 3 but for $\left(P_{c^{\prime }}+P_{c^{\prime }}^{*}\right)=1$ in the TCP-score (3). Final fraction $f_{f}^{\mathrm{USD}}$ of countries with a trade currency preference for USD versus the initial fraction $f_{i}^{\mathrm{USD}}$ of these countries for years 2010 (left panel) and 2019 (right panel). The number of final states and their corresponding value $f_{f}^{\mathrm{USD}}$ are initial fraction $f_{i}^{\mathrm{USD}}$ dependent. The color of the points represents the ratio of the Monte Carlo process with the corresponding final state $\rho_{f_{f}^{\mathrm{USD}}}\left(f_{i}^{\mathrm{USD}}\right)$, low ratio in cold blue and high ration in violet. The central panel shows the evolution of $\rho_{f_{f}^{\mathrm{USD}}}$ with $f_{i}^{\mathrm{USD}}$. Each line corresponds to a given $f_{f}^{\mathrm{USD}}$. Full (empty) circles and solid (dashed) lines correspond to the year 2019 (2010).

Finally, we make a note about the asynchronous Monte Carlo iteration process controlled by the TCP-score (3). This score computed for a given country *c* contains not only trade flows between the country *c* and its trade partners $c^{\prime }$ given by the $S_{c^{\prime }c}$ matrix elements, but also the factor $\left(P_{c^{\prime }}+P_{c^{\prime }}^{*}\right)$, which is the relative economical weight of the countries *c* direct trade partners. The more important a country is in the world trade network, the higher its relative economical weight given by its ImportRank and ExportRank. In a sense, this last factor takes into account the world importance of a country $c^{\prime }$. This is similar to the opinion formation approach used in [15], for which the position of the neighbors of an agent in the society elite [1] is taken into account to infer the opinion of this agent. Thus, the *vote* of China or the USA counts in the choice of the TCP of their direct partners much more than the *vote* of the least developed countries. We think that this factor correctly takes into account the trade relations between the countries. However, in the case of one object, the use of this factor in the TCP-score, we also present, in Figure A5, Figure A6, and Figure A7, the results when this factor is neutralized by taking $P_{c^{\prime }}+{P_{c^{\prime }}}^{*}=1$ in (3). In such a case, there is a larger number of steady state fractions $f_{f}^{\mathrm{USD}}$, as shown in Figure A7. The distribution of the swing countries is shown in Figure A5 for year 2010 and 2019 and with a high fraction $f_{i}^{\mathrm{USD}}=0.9$ of countries initially preferring USD. Qualitatively, we still observe a significant increase of the BRI group from 2010 to 2019, but certain countries remain in the swing group in 2019, i.e., Southeast Asia, Central Asian countries, South Korea and Japan in Asia, a few West African countries and Madagascar in Africa, and Chile and Peru in South America. The probability that a country adopt a TCP for USD at the final stage of the asynchronous Monte Carlo iterations is shown, for $f_{i}^{\mathrm{USD}}=0.9$, in Figure A6. Here, many of the swing countries from Figure A5 adopt a USD TCP in 2010 and 2019. Rather naturally, the steady state features are more stable and robust when the weight $P_{c^{\prime }}+P_{c^{\prime }}^{*}$ of the countries belonging to the trade leader elite is taken into account in the TCP-score (3), and hence, we present our analysis in the frame of this relation.

## 4. Regomax for the Anglo-Saxon Group and the BRICS+

**Figure 5:**
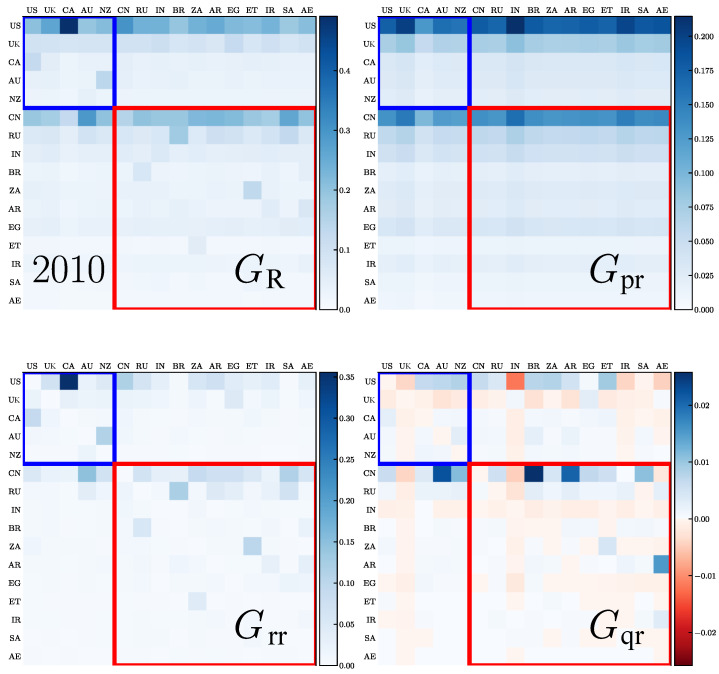
Reduced Google matrix $G_{R}$ and its components for the ANGL countries and the BRICS+ and for the year 2010: $G_{R}$ (**top left**), $G_{\mathrm{pr}}$ (**top right**), $G_{\mathrm{rr}}$ (**bottom left**) and $G_{\mathrm{qr}}$ (**bottom right**). For the $G_{\mathrm{qr}}$ matrix, the relative weight of negative elements is $\left(W_{+}-W_{-}\right)/\left(W_{+}+W_{-}\right)=0.31$, where $W_{+}$ and $W_{-}$ are, respectively, the mean of positive and negative elements (in absolute value).

**Figure 6:**
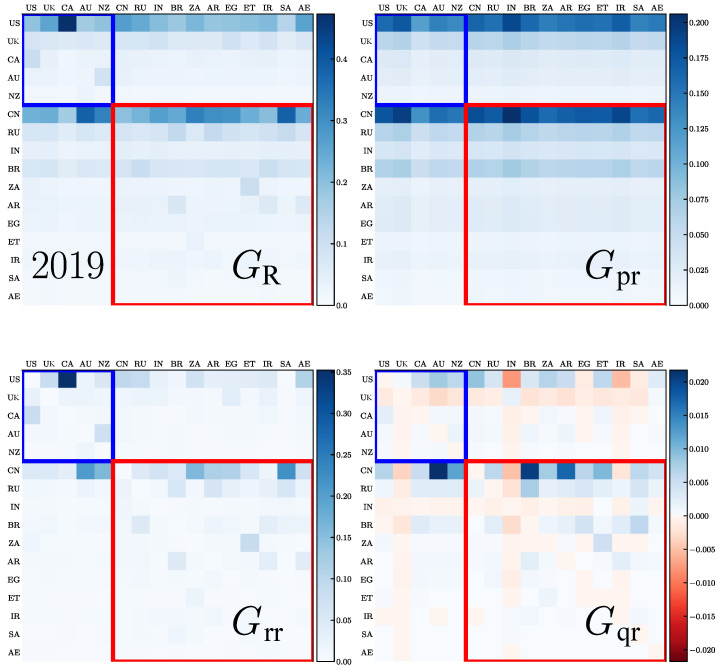
Same as in Figure 5 but for the year 2019; here, $\left(W_{+}-W_{-}\right)/\left(W_{+}+W_{-}\right)=0.27$.

We present the REGOMAX description of the trade interactions between the 5 Anglo-Saxon countries (ANGL group) and the 11 BRICS+ countries obtained from the 2010 and 2019 WTN. The whole Google matrix *G* of the WTN has a size of N=194 that is the total number of countries. As described in Section 2.2, the REGOMAX algorithm allows for obtaining the reduced Google matrix $G_{R}$ of size $N_{R}=16$, which describes the trade interactions between these 16 countries taking into account all their indirect interactions via the other 194−16=178 countries of the global WTN. Thus, $G_{R}$ and its components characterize the integrated import flows. The matrix $G_{R}$ has three components, $G_{R}=G_{\mathrm{pr}}+G_{\mathrm{rr}}+G_{\mathrm{qr}}$. All these matrices, $G_{R}$, $G_{\mathrm{rr}}$, $G_{\mathrm{pr}}$, $G_{\mathrm{qr}}$, are shown in Figure 5 and Figure 6 for the years 2010 and 2019, respectively. In each core group, ANGL and BRICS+, the countries are sorted according to their values of ImportRank $P_{c}$ and ExportRank $P_{c}^{*}$, namely, by descending order of the $\max_{c}\left(P_{c},P_{c}^{*}\right)$ value. We remind the reader that usually $G_{\mathrm{pr}}$ is close to a matrix composed by columns filled with the PageRank vector components of the $N_{R}$ countries, $G_{\mathrm{rr}}$ encodes the direct transitions between the $N_{R}$ countries and $G_{\mathrm{qr}}$ is obtained by the sum over all the indirect pathways linking the $N_{R}$ through the whole global WTN. By definition, the weight of the $G_{R}$ components are $W_{R}=\sum_{ij}G_{R}\left(i,j\right)/N_{R}=1$, and thus, $W_{R}=W_{\mathrm{rr}}+W_{\mathrm{pr}}+W_{\mathrm{qr}}=1$, where the weights of the three matrix components are defined in the same way as for $G_{R}$. For Figure 5 and Figure 6, we have $W_{\mathrm{pr}}=0.723$ and 0.695, $W_{\mathrm{rr}}=0.259$ and 0.287, $W_{\mathrm{qr}}=0.018$ and 0.018, respectively for the years 2010 and 2019. Thus, the main contribution to $G_{R}$ comes from $G_{\mathrm{pr}}$ and $G_{\mathrm{rr}}$, while the contribution of $G_{qr}$, although small, provides important information on indirect trade transfers between the $N_{R}$ countries.

From Figure 5 and Figure 6, we see that the strongest transition elements form two horizontal lines for the USA and China that are well visible in $G_{R}$ and $G_{\mathrm{pr}}$. This is rather natural since these two countries are at the top of PageRank probabilities and are the most important importers in world trade. According to reduced Google matrix $G_{R}$, the most strong trade import to USA is from Canada, while for China, it is from Australia and Saudi Arabia. We also see that the total trade transfer from the 16 countries towards the USA ($T_{\mathrm{US}}=3.9$ in 2010 and 3.2 in 2019) and towards China ($T_{\mathrm{CN}}=3.6$ in 2010 and 4.2 in 2019) is significantly changed from 2010 to 2019, with it being enhanced for China and decreased for the USA (here, $T_{c^{\prime }}=\sum_{c}{G_{R}}_{c^{\prime },c}$, where $c^{\prime }$ is either the USA or China, and the sum is performed over the countries *c* different from the country $c^{\prime }$).

From the $G_{\mathrm{pr}}$ matrix, we also see that the USA and China have important trade import from the UK, India and, surprisingly, from Iran, both in 2010 and 2019.

The contributions from the direct trade are given by the matrix $G_{\mathrm{rr}}$ components. For the USA, in 2010, the strongest imports are from Canada and from China (but significantly smaller), and in 2019, it is still Canada and the Arab Emirates. For China, the strongest imports are from Australia, South Africa and Saudi Arabia in 2010 and 2019 (New Zealand also becomes important in 2019).

The indirect trade transitions are described by the matrix $G_{\mathrm{qr}}$ elements. These matrix elements are on average smaller than those of the matrices $G_{\mathrm{pr}}$ and $G_{\mathrm{rr}}$; however, they allow for the establishment of indirect relations between countries. Thus, for the USA, we have the most significant matrix elements from India, Arab Emirates, Iran and the UK in 2010, and India and Iran in 2019—these elements are negative. In principle, negative elements for the $G_{\mathrm{qr}}$ matrix are not forbidden since only the $G_{R}$ elements should be positive. Usually, negative elements are small and appear only for $G_{\mathrm{qr}}$ (see, e.g., [36]). Here, the negative elements are well visible even if they still remain smaller compared to the positive ones. Such a negativity implies that for the USA, the indirect trade links between the USA and India, or Iran, reduce the direct trade transfers to the USA from these countries. For China, the strongest positive indirect links are from Australia, Brazil and Argentina both in 2010 and 2019; negative elements are small (the most visible is a negative link from India in 2019).

It is possible to consider the REGOMAX results in more depth, or to apply them to other groups of countries, but we will not opt for such extensions since the main objective of this work is an analysis of the opinion formation in the WTN.

**Figure 7:**
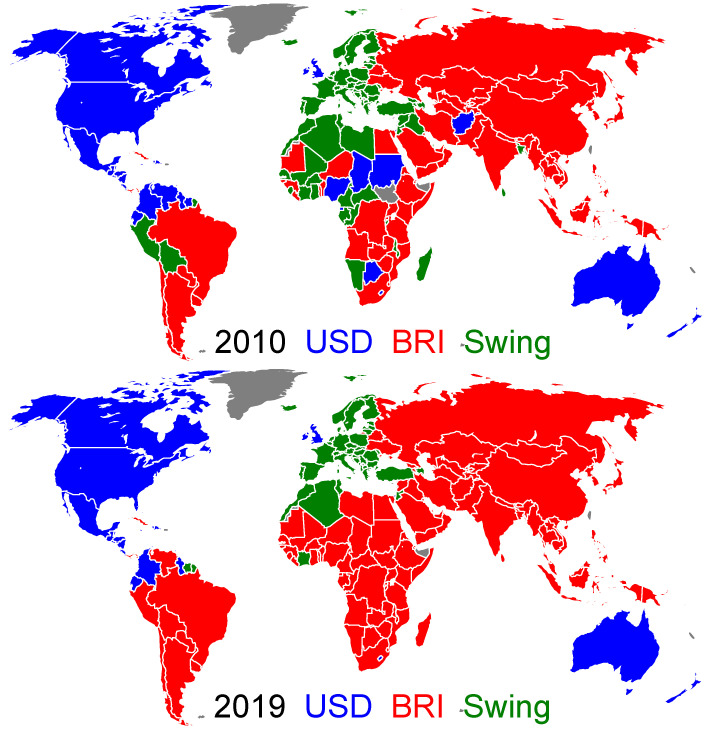
Same as Figure 1, but for when the ImportRank and ExportRank probabilities in the TCP-score (3) are replaced by PageRank and CheiRank probabilities of the WTN Google matrix.

Finally, we note that in the TCP-score (3), the probabilities can be defined not from the trade volume fractions of export and import, i.e., from the ImportRank and the ExportRank, but from the PageRank and CheiRank probabilities of the global Google matrix *G* constructed from the trade of 194 countries. The countries’ TCPs obtained with such PageRank and CheiRank probabilities implemented in the TCP-score (3) for 2010 and 2019 are shown in Figure 7. They are very close to those of Figure 1 obtained with ImportRank and ExportRank probabilities. This shows that the obtained results are very robust and are not significantly affected by moderate modifications of the measure of the global importance of the export and export capabilities of a country we use in the TCP-score definition (3).

## 5. Three Currencies Case

Let us now consider three currencies, namely, USD, EUR and BRI. We keep the USD and the BRI core groups, namely, the ANGL group and the BRICS+ (see Table 1). The EUR core group is the EU9 group constituted by Austria, Belgium, France, Germany, Italy, Luxemburg, Netherlands, Portugal and Spain [32]. Hence, the countries of the ANGL group keep trading in USD, the countries of the EU9 group in EUR and the BRICS+ in BRI. For each country *c*, we compute the following TCP-score for each currency $¢\in \left\{\mathrm{USD},\mathrm{EUR},\mathrm{BRI}\right\}$

(4) $$Z_{c,¢}=\frac{\sum_{c^{\prime }\neq c}^{\left(¢\right)}\left(S_{c^{\prime }c}+S_{c^{\prime }c}^{*}\right)\left(P_{c^{\prime }}+P_{c^{\prime }}^{*}\right)}{\sum_{c^{\prime }\neq c}\left(S_{c^{\prime }c}+S_{c^{\prime }c}^{*}\right)\left(P_{c^{\prime }}+P_{c^{\prime }}^{*}\right)},$$

where the sum $\sum_{c^{\prime }\neq c}^{\left(¢\right)}$ is performed over all the countries who are commercial partners of the country *c* and who prefer to trade with the currency *¢*. For a given country *c*, the sum of these scores over the different considered currencies is equal to one, i.e., $\sum_{¢}Z_{c,¢}=1$. The country *c* then adopts the trade currency for which the TCP-score is the maximum, i.e., ${¢}_{1}$ such as $Z_{c,{¢}_{1}}=\max_{¢}Z_{c,¢}$. The asynchronous Monte Carlo procedure (see Section 2.1) is performed with the above defined TCP-score (4). For a given year among those considered (2010–2020), the final steady state distribution of the TCPs is unique and does not depend on the initial distribution of the TCPs. By contrast with the two currencies model (see Section 3), there is no swing country for whom the TCP changes according to the initial distribution of the TCPs.

**Figure 8:**
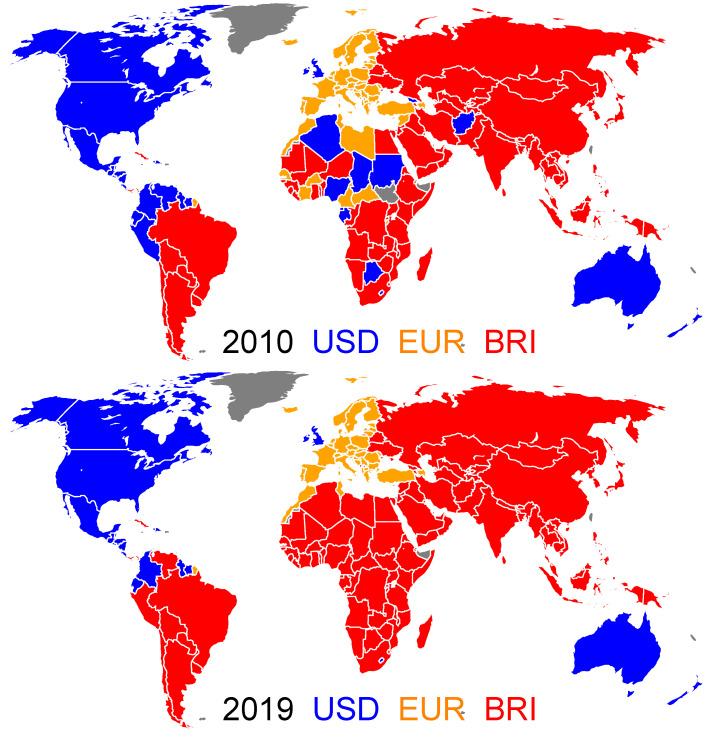
World distribution of the trade currency preferences for the years 2010 (**top**) and 2019 (**bottom**). The countries belonging to the USD, EUR and BRI groups are colored in blue, gold and red. Countries colored in gray have no trade data reported in the UN Comtrade database for the considered year [21].

The world distributions of the TCPs for the years 2010 and 2019 are shown in Figure 8. We observe that both in 2010 and 2019, the USD group gathers, beside the ANGL group countries, the Central American countries and some of the countries of the northern part of South America; the EUR group gathers, besides the EU9 group countries, the European countries delimited to the east by the NATO border; the BRI group gathers, beside the BRICS+, most of the Asian and South American countries. The main difference between the 2010 and 2019 years is the situation of the African continent. Although this continent appears fragmented in 2010, echoing the post-1989 era battle of influence between France, the USA, Russia and China, in 2019, the whole African continent appears to be under the influence of the BRI with the exception of Morocco and Tunisia. By comparison with the two currencies model (see Section 3), the introduction of a third currency, namely, the EUR, crystallizes the swing group: undecided countries in the two currencies model now adopt a fixed currency preference independent of the initial distribution of the TCPs. Moreover, the new EUR group mostly corresponds to the swing group (see Figure 2).

**Figure 9:**
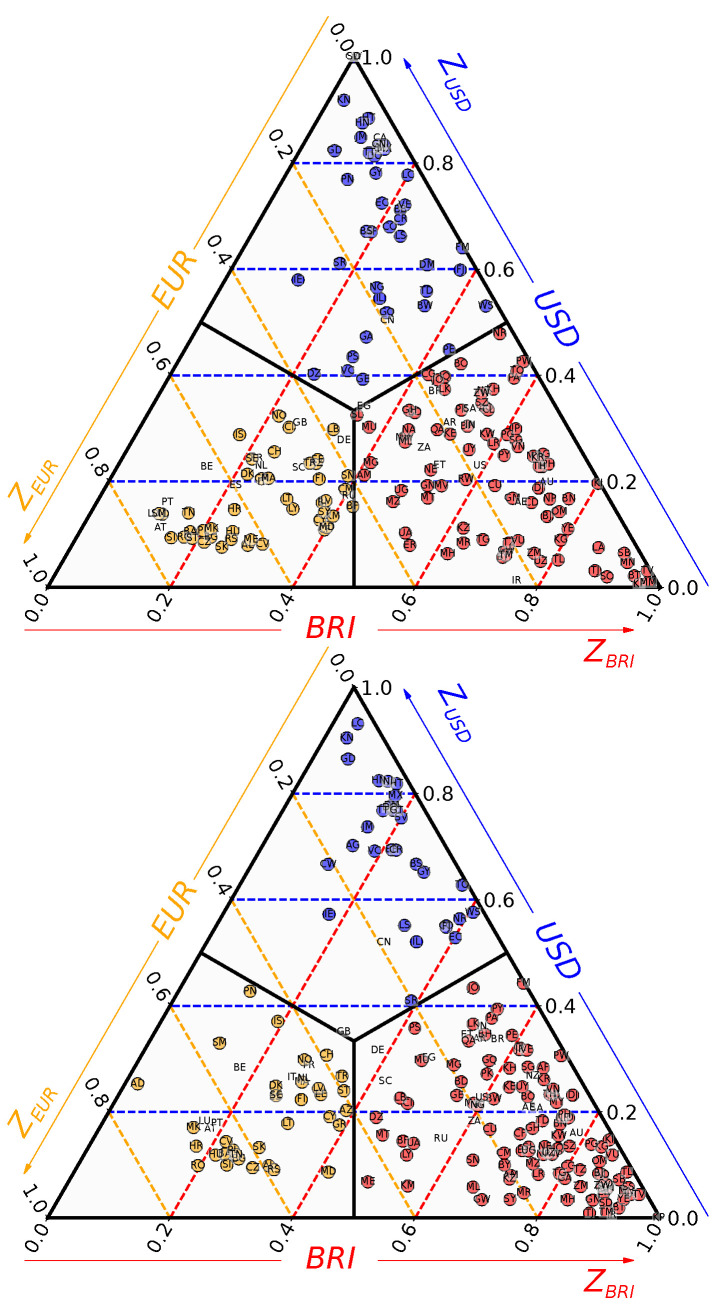
Distribution of countries’ TCP scores $\left(Z_{\mathrm{USD}},Z_{\mathrm{EUR}},Z_{\mathrm{BRI}}\right)$ for 2010 (**top**) and 2019 (**bottom**). A country is represented by a circle. Colors are associated with TCPs: blue for USD, gold for EUR and red for BRI. The $Z_{\mathrm{USD}}$ coordinate is read along the dashed blue horizontal lines, the $Z_{\mathrm{EUR}}$ coordinate along the gold dashed oblique lines and the $Z_{\mathrm{BRI}}$ coordinate along the red dashed oblique lines.

The distribution of the TCP-scores (4) over the range $\left[0,1\right]\times \left[0,1\right]\times \left[0,1\right]$ is shown in Figure 9. From 2010 to 2019, we observe a clear depletion of the USD and the EUR sectors to the profit of the BRI sector. Whereas some countries are strongly influenced by USD or BRI with $Z_{\mathrm{USD}}>0.8$ or $Z_{\mathrm{BRI}}>0.8$, there is not such a country with $Z_{\mathrm{EUR}}>0.8$. Consequently, the countries with a TCP for EUR are moderately bounded to it, and a non-negligible part of them are on the brink of a transition towards the BRI sector. Moreover, in 2019, the countries in the USD and BRI sectors are principally located in the $Z_{\mathrm{EUR}}<0.2$ sector, indicating that the countries with a TCP for USD or BRI are very weakly influenced by the EUR.

**Figure 10:**
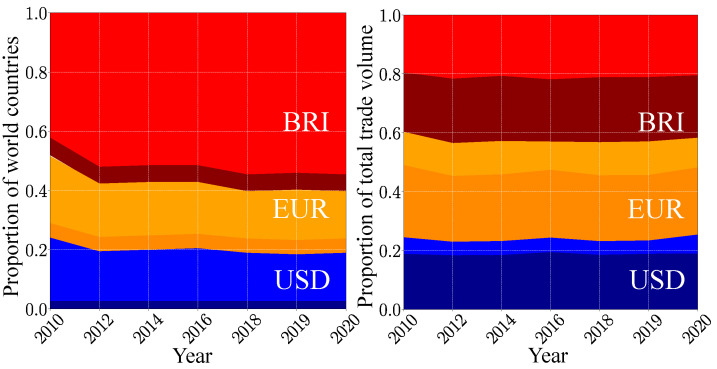
Time evolution of the size of the trade currency preference groups. The width of a given band corresponds to the corresponding fraction of world countries in a TCP group (**left panel**) and to the corresponding fraction of the total trade volume generated by this group (**right panel**). The USD group is colored in blue, the BRI group in red and the EUR group in gold. Within the BRI (USD) [EUR] group, the proportion corresponding to the BRICS+ (the ANGL countries) [the EU9 group] is shown in dark red (dark blue) [dark gold].

The time evolutions of the USD, EUR and BRI groups, shown in Figure 10, share strong similarities with those of the USD, BRI and swing groups for the two currencies model (Figure 4). Again, the swing group is replaced by the EUR group. We also note that the results obtained in the frame of the three currencies model are very similar if one considers the old BRICS organization with the five historic partners [31] or the current BRICS+ organization.

**Figure A8:**
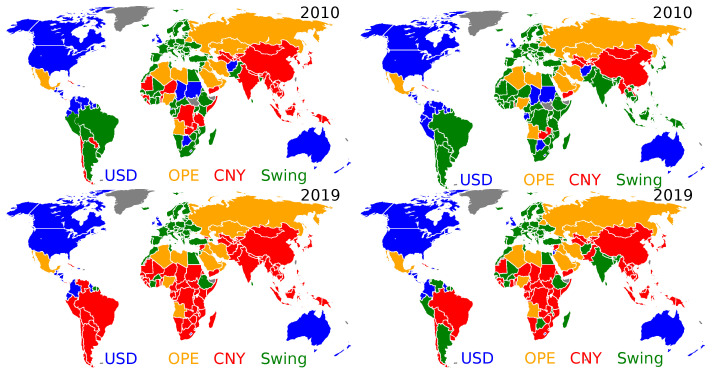
World distribution of the trade currency preferences for the years 2010 (**top**) and 2019 (**bottom**). The price of oil and gas products is multiplied by K=1 (**left**) and K=4 (**right**). The countries belonging to the USD group, the CNY group, and the OPE group are colored in blue, red, and gold, respectively. Those belonging to the swing group are colored in green. Countries colored in gray have no trade data reported in the UN Comtrade database for the considered year [21].

**Figure A9:**
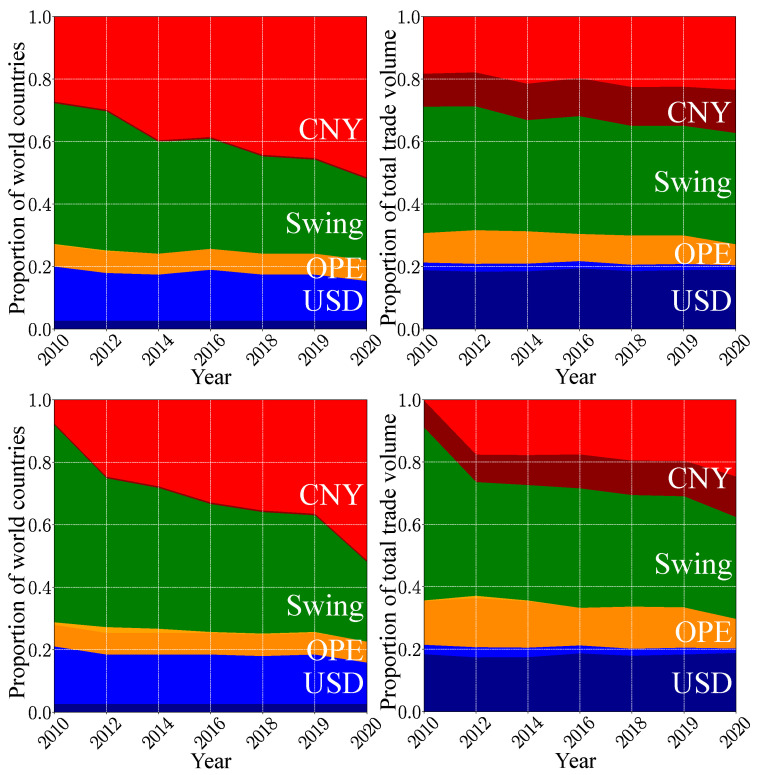
Time evolution of the size of the trade currency preference groups. The width of a given band corresponds to the corresponding fraction of world countries in a TCP group (**left panel**) and to the corresponding fraction of the total trade volume generated by this group (**right panel**). The price of oil and gas products is multiplied by K=1 (**top**) and K=4 (**bottom**). The USD group is colored in blue, the CNY group in red, the OPE group in gold, and the swing group in green. Within the USD (CNY) [OPE] group, the proportion corresponding to the Anglo-Saxon countries (China) [OPEC+ countries] is shown in dark blue (dark red) [dark gold].

As a summary, our currency model allows for probing on how influential economical blocks of countries in the frame of international trade are. Another example of application is, e.g., how influential the OPEC+ countries are if the price of oil and petroleum products are multiplied by a factor *K*. In this last example, we keep the Anglo-Saxon countries trading in USD, China in CNY and 11 OPEC+ countries (listed in Table 1) in OPE, a hypothetical currency. Contrarily to the hypothetical BRICS currency, no political entity claims the creation of such a petrocurrency. Nonetheless, in our model, a country with a TCP for OPE will have significant trade ties with the OPEC+ countries. Once the steady state of the asynchronous Monte Carlo designation of the countries’ TCPs is achieved, we obtain four groups (see Figure A8): the USD group, the CNY group, the OPE group and a swing group. This latter group contains countries that, depending on the initial distribution of the TCPs, swing between the three currencies, namely, USD, CNY and OPE. Besides the fact already mentioned through the BRICS+ case that China gained a prominent economical influence during the last decade, we observe that the OPEC+ is not influential enough to constrain extra-OPEC+ countries to always adopt OPE as TCP, i.e., whatever the initial TCP is distribution over the countries. Nonetheless, the influence of the OPEC+ countries operates through the swing countries that possibly can adopt OPE as a TCP. For the year 2010, the number of swing countries significantly increases if one multiplies the price of oil and gas products by K=4 (Figure A8): India and South-East Asian countries becomes swing countries no longer, exclusively preferring CNY as trade currency. This increase of the number of swing countries with the factor K=4 fades in 2019. From Figure A9, we still observe during the last decade the significant growing of the ability of China’s economy to gather in the CNY *club*, with many countries trading a non-negligible part of the global trade volume, whereas the USD group captures a moderate number of extra-ANGL countries but with a modest trade volume, and the OPE group is unable to capture extra-OPEC+ countries. Besides the mechanical increase of the OPEC+ traded volume, the multiplication of the oil and gas products prices by a factor K=4 induces, from 2010 to 2019, a larger number of countries belonging to the swing group in comparison with the K=1 case. As the OPE and USD groups are similar for the K=1 and K=4 cases, the CNY group less efficiently captures countries in the case of expensive oil and gas. In 2020, the situation is different, as the size of the CNY group is the same for the two cases, with it being insensitive to the increase of oil and gas products’ prices.

## 6. Conclusions

In this work, we extend and generalize the approach of opinion formation to the WTN, which represents an example of a complex directed network. Our method is based on asynchronous Monte Carlo iterations of local spins, for which the orientation describes the trade preference of a given country to perform trade with one or another currency. We consider two or three core groups of countries that always keep trading with a certain currency, e.g., USD, BRI or EUR, and then we follow the Monte Carlo evolution for spin orientations that converge to a final steady state orientation of spins (opinions or trade currency preferences of the countries). The local orientation of spins/opinions, namely, the trade currency preference of a country *c*, is determined by the value of a TCP-score, which is a linear combination of terms encoding both the relative importance of the volume exchanged between the country *c* and its economical partners, and the relative importance of the country *c* economic partners. We note that a somewhat similar Monte Carlo iterations process with a certain linear condition for spin orientations has also been considered in problems of associative memory (see, e.g., [29,30]).

We mainly consider the case of the currency battle between 5 Anglo-Saxon countries with a fixed TCP for USD and the 11 BRICS+ countries with a fixed TCP for the hypothetical BRI currency. The obtained results, based on the WTN constructed from the UN Comtrade database [21] for the years 2010–2020, show the existence of two fixed groups of countries with a firm TCP for USD and BRI (these groups do not depend on the initial fractions of the countries with initial TCP for USD or BRI) and a group of swing countries that may change their TCP depending on the initial fraction of countries with TCP for USD or BRI. We show that from the year 2016, the BRI group contains more than 50% of the world’s countries. Thus, in the year 2020, the BRI group contains 58% of all the world countries, the USD group 16% and the swing group 26%.

We also describe the reduced Google matrix analysis of the WTN, showing that it establishes integrated trade relations between Anglo-Saxon and BRICS+ countries.

Finally, we extend the model to three currencies. On one hand, we consider the currencies USD, BRI and EUR, which are supported by nine EU countries. In this case, the TCP distribution, for a given year, converges toward a unique solution that is independent of the initial random world distribution of the TCPs. By comparison with the two currencies model, roughly speaking, most of the swing countries adopt the EUR as trade currency, whereas the USD and the BRI groups behave similarly within the two models. On the other hand, in order to analyze the economic influence of the OPEC+ countries, we introduce a fictitious petrocurrency, OPE, which competes against USD and CNY.

As far as we know, the use of an opinion formation model to probe the economic influence of currencies and/or economic organizations within international trade is novel. Our model is based solely on the structure of the WTN, namely, the bilateral trade flows between world countries, and does not take into account any political or geopolitical aspects. As a conclusion, we state that nowadays, the structure of international trade will clearly favor the emergence of the hypothetical BRI currency pegged to the BRICS+ economies.

## Figures and Tables

**Table 1:** List of countries belonging to the core groups: the Anglo-Saxon group (ANGL), the BRICS+, the kernel of 9 European countries (EU9), and the OPEC+ countries (we select those producing more than 1 million oil barrels in 2022). The countries are sorted in decreasing order of max$\left(P_{c},P_{c}^{*}\right)$, where $P_{c}$ and $P_{c}^{*}$ are the ImportRank and the ExportRank of a country *c* in 2019 WTN, respectively.

Core Group	Country Name	Currency
Anglo-Saxon	United States of America	USD
	United Kingdom	
	Canada	
	Australia	
	New Zealand	
BRICS+	China	BRI
	India	
	Russia	
	United Arab Emirates	
	Brazil	
	Saudi Arabia	
	South Africa	
	Argentina	
	Egypt	
	Iran	
	Ethiopia	
EU9	China	EUR
	Germany	
	France	
	Netherlands	
	Italy	
	Belgium	
	Spain	
	Austria	
	Portugal	
	Luxembourg	
OPEC+	Saudi Arabia	OPE
	Russia	
	Iraq	
	United Arab Emirates	
	Kuwait	
	Iran	
	Mexico	
	Kazakhstan	
	Angola	
	Nigeria	
	Oman	
	Algeria	
	Libya	

## Data Availability

The data presented in this study are available on request from the corresponding author. The world trade data are available at https://comtrade.un.org (accessed on 30 December 2023).

## Abbreviations

The following abbreviations are used in this manuscript:

ANGL	Core group of Anglo-Saxon countries
BRI	Hypothetical currency pegged to the BRICS economies
BRICS	Brazil, Russia, India, China, South Africa
BRICS+	Extended BRICS
CNY	Chinese yuan
EU	European Union
EUR	Euro
NATO	North Atlantic Treaty Organization
OPE	Hypothetical currency pegged to the major oil and gas country producers
OPEC	Organization of the Petroleum Exporting Countries
OPEC+	Extended OPEC
REGOMAX	Reduced Google matrix
TCP	Trade currency preference
UK	United Kingdom
UN	United Nations
USD	US Dollar
WTN	World Trade Network
WWW	World Wide Web
